# Towards Inclusive Colorectal Cancer Screening: Experiences and Needs of Adults With Intellectual Disabilities

**DOI:** 10.1002/cam4.71212

**Published:** 2025-09-04

**Authors:** Theresa Wagner, Alma R. Herscovici, Amelie Fuchs, Sebastian Kabas, Mara Hilbert, Laura M. König, Matthias Unseld, Elisabeth L. Zeilinger

**Affiliations:** ^1^ Department of Clinical and Health Psychology, Faculty of Psychology University of Vienna Vienna Austria; ^2^ Division of Hematology and Hemostaseology, Department of Medicine I Medical University of Vienna Vienna Austria; ^3^ Vienna Doctoral School in Cognition, Behavior and Neuroscience University of Vienna Vienna Austria; ^4^ Department of Clinical Research SBG Academy for Ageing Research, Haus der Barmherzigkeit Vienna Austria

**Keywords:** colorectal cancer screening, early detection of cancer, healthcare needs, inclusion, intellectual disabilities, qualitative research, thematic analysis

## Abstract

**Introduction:**

People with intellectual disabilities (ID) face significant barriers to healthcare and preventive cancer care, resulting in delayed cancer diagnosis and higher mortality rates. There is limited understanding of the factors that influence their participation in colorectal cancer (CRC) screening, particularly from their own perspectives. This study aimed to identify the barriers, facilitators, and needs of people with ID for an inclusive CRC screening programme from their own experiences and viewpoints.

**Methods:**

Semi‐structured qualitative interviews and focus groups (*N* = 31) were conducted with adults with ID in Austria. Interviews and group discussions were audio recorded and transcribed verbatim. Thematic analysis was used as a flexible method to analyse the data.

**Results:**

Five themes were identified from the data with each consisting of two to four sub‐themes: (1) independence within individually adjusted scopes of arrangement and decision‐making, (2) ‘When it comes to health, I do it’, (3) enhancing wellbeing, (4) seeing the person first, then their ID, and (5) deficits in resources and the healthcare system.

**Conclusion:**

The findings reveal significant barriers to healthcare and preventive cancer care for people with ID. The following practical implications were derived: Eliminating discrimination, improving accessibility, designing appropriate information and educational materials, implementing mandatory ID‐specific training for health professionals, considering the importance of emotions and implementing ID‐appropriate health services. Considering these aspects when developing inclusive cancer screening programmes is of paramount importance to promote equitable health and cancer prevention, especially for marginalised and vulnerable groups.

## 1 Introduction

Cancer is a leading cause of death worldwide, with colorectal cancer (CRC) being the second leading cause [1]. People with intellectual disabilities (ID) face an increased risk of developing CRC due to several cancer risk factors, including overweight, unhealthy diet and physical inactivity [2, 3, 4]. Accelerated ageing in this group increases susceptibility to early‐onset cancers [5] and the clinical presentation of CRC can be masked by challenging behaviour and communication difficulties, leading to delayed diagnosis and higher CRC mortality rates [3, 6, 7, 8]. Furthermore, the similarity of CRC symptoms to those of common chronic or minor abdominal diseases underlines the importance of objective testing, particularly for cancers of the lower gastrointestinal tract [8]. Faecal occult blood tests (FOBT), faecal immunochemical tests (FIT) or colonoscopy are options for objective testing. Although cancer screening is an effective method of early detection and reducing mortality, people with ID are significantly less likely to be screened for CRC [7, 9, 10, 11]. Studies investigating factors associated with the uptake of CRC screening are scarce [9], and there is a paucity of research conducted from the perspective of people with ID themselves [12, 13].

ID is classified as Disorders of Intellectual Development by *the International Classification of Diseases, 11th Revision* (ICD‐11). ID comprises a wide range of conditions with various causes that originate during the developmental period and are defined by significantly subaverage intellectual functioning (two or more standard deviations below the mean) and adaptive behaviour (expressed in conceptual, social and practical skills) [14, 15]. ID is associated with major health inequalities in access to healthcare. The observed mortality disparities and underuse of cancer screening not only emphasise adverse inequalities, but also indicate that screening programmes are not adequately addressing the needs of people with ID.

Article 25 of the United Nations Convention on the Rights of People with Disabilities (UN‐CRPD), calls for the ‘highest attainable standard of health’ and ‘ensuring access to health services for people with disabilities’ [16]. To achieve this, it is crucial to address inequalities in access to cancer screening services and to implement inclusive organised screening programmes. As there is a significant lack of research on barriers and facilitators to CRC screening from the perspective of people with ID themselves, those most affected are left out of the discussion and without a voice.

The present study uniquely incorporates the views of people with ID, offering first‐hand insights through interviews and focus groups, addressing a critical gap in existing literature. Conducted in Austria, where there is yet no national CRC screening programme, this study aims to inform the development of inclusive screening programmes to support appropriate and equitable preventive health measures for people with disabilities. Similar to Austria, inclusive screening programmes are lacking in many other countries. The results of this study could inform the development of such programmes and be transferred to other countries and contexts. Therefore, the aim of this study was to identify the barriers, facilitators, and needs that influence participation in CRC screening from the perspective of people with ID using a qualitative approach based on interviews and focus groups.

## 2 Materials and Methods

### 2.1 Research Design

Data collection involved semi‐structured qualitative interviews and focus groups, based on participants' preferences, enabling individuals with ID to share their insights as experts for themselves. Language level A1–A2 has been carefully used in all materials and conversations. As appreciation for their participation in the study, the participants received a 20€ voucher. The study was pre‐registered on the Open Science Framework (OSF; https://osf.io/ndfx3).

### 2.2 Participant Recruitment

The recruitment strategy was designed using a maximum variation sampling method to collect comprehensive and diverse data. Various organisations, accommodation providers, self‐advocacy groups, and day centres for people with ID in all nine Austrian federal states were contacted by email, and all received the same invitation flyer, written in easy language (language level A1–A2) and accessible to people with ID. The invitation flyer was designed by the research team and included information about the topic, duration, moderators and contact details, and was reviewed by a team member trained for easy language. Inclusion criteria for participation were having an intellectual disability, being able to express themselves verbally, and being 18 years of age or older. The age of inclusion was set at 18 years as there is no organised programme or guidelines for age limits in Austria, and stool tests should be performed as part of the annual health check‐up from the age of 18 years. To identify the barriers, facilitators and needs, a broader age range provides the opportunity to gain a wider range of perspectives. Once diversity in the data was achieved and saturation was reached, recruitment was stopped. Recruitment and data collection started in April 2023 and ended 6 months later. Ethical approval was obtained from the Ethics Committee of the Medical University of Vienna (No. 2125/2022). Informed consent procedures were adapted for accessibility, using easy language and visual aids to facilitate understanding. Participants were given the option to withdraw at any time without consequences. Additionally, measures were taken to ensure psychological safety, particularly given the sensitive nature of discussing health and cancer screening. A clinical psychologist could be contacted at any time after participation if needed.

### 2.3 Data Collection

The guideline for focus groups and interviews followed a semi‐structured qualitative format with open‐ended questions, images, and a fictional case study featuring an individual with ID [17, 18, 19, 20]. The File S1 shows the topics covered and the material used. Easy language, pictures and pictograms were used to aid communication. The guideline was developed by experts with a psychological background and experience in ID, based on published literature [21, 22, 23, 24, 25] and pilot interviews. Both interviews and focus groups used the same guideline, adapted for each format. Interviews averaged 45 min, while focus groups lasted 60–90 min. Most sessions were conducted face‐to‐face in familiar environments, with one focus group held online. Only participants and researchers were present and part of the discussions. A support person was present in three focus groups, but care was taken to ensure that they remained in the background and did not influence the group discussions. Data collection was conducted by four trained team members having a psychological background, with one interviewer/moderator and a note‐taker present at each session. The interviews were audio recorded and transcribed verbatim.

### 2.4 Data Analysis

Thematic analysis was conducted following an inductive, data‐driven, latent and constructivist approach. To develop an initial, but comprehensive coding scheme, one interview and one focus group, reporting a wide range of experiences, were jointly coded by all members of the coding team (TW, ARH, MH, SK). Two more interviews and one focus group were coded independently and discussed. After reorganising the coding scheme, the remaining interviews and focus groups were coded individually by three researchers. Uncertainties were discussed and clarified. Once the final coding system had been applied to all interviews and focus groups, the codes were clustered, and potential themes were discussed by the coding team, the lead researcher (ELZ) and an experienced qualitative researcher (LMK). Reviewed themes were defined and named. The analysis was conducted using MAXQDA (2022) software. The coding team had a psychological background with minimal preconceptions prior to data collection and analysis to avoid bias, while the lead researcher brought an experienced perspective.

## 3 Results

### 3.1 Study Sample

The final sample consisted of 31 persons with ID (9 female) aged 18–85 years (M = 48.8, SD = 18.8; Mdn = 49) residing across five of Austria's nine federal states. We conducted five individual interviews and five focus groups, each with three to nine participants. The sample covered a wide range of living situations, different levels of ID, and included self‐advocates (see Table 1).

**TABLE 1:** Sociodemographic characteristics of participants.

	*N* = 31		
		*n*	%
Gender	Male	22	71.0
	Female	9	29.0
Living area	Urban	18	58.1
	Rural	13	41.9
Living situation	Fully cared for	12	38.7
	Semi‐independent	7	22.6
	With family	5	16.1
	Alone/with partner	5	16.1
	Missing	2	6.5
Legal guardianship^a^	Yes	13	41.9
	No	9	29.0
	Missing	9	29.0

*Note:* Focus group participants = 26, interview participants = 5.

^a^ People with ID have someone to make decisions and act on their behalf.

### 3.2 Qualitative Results

**FIGURE 1:**
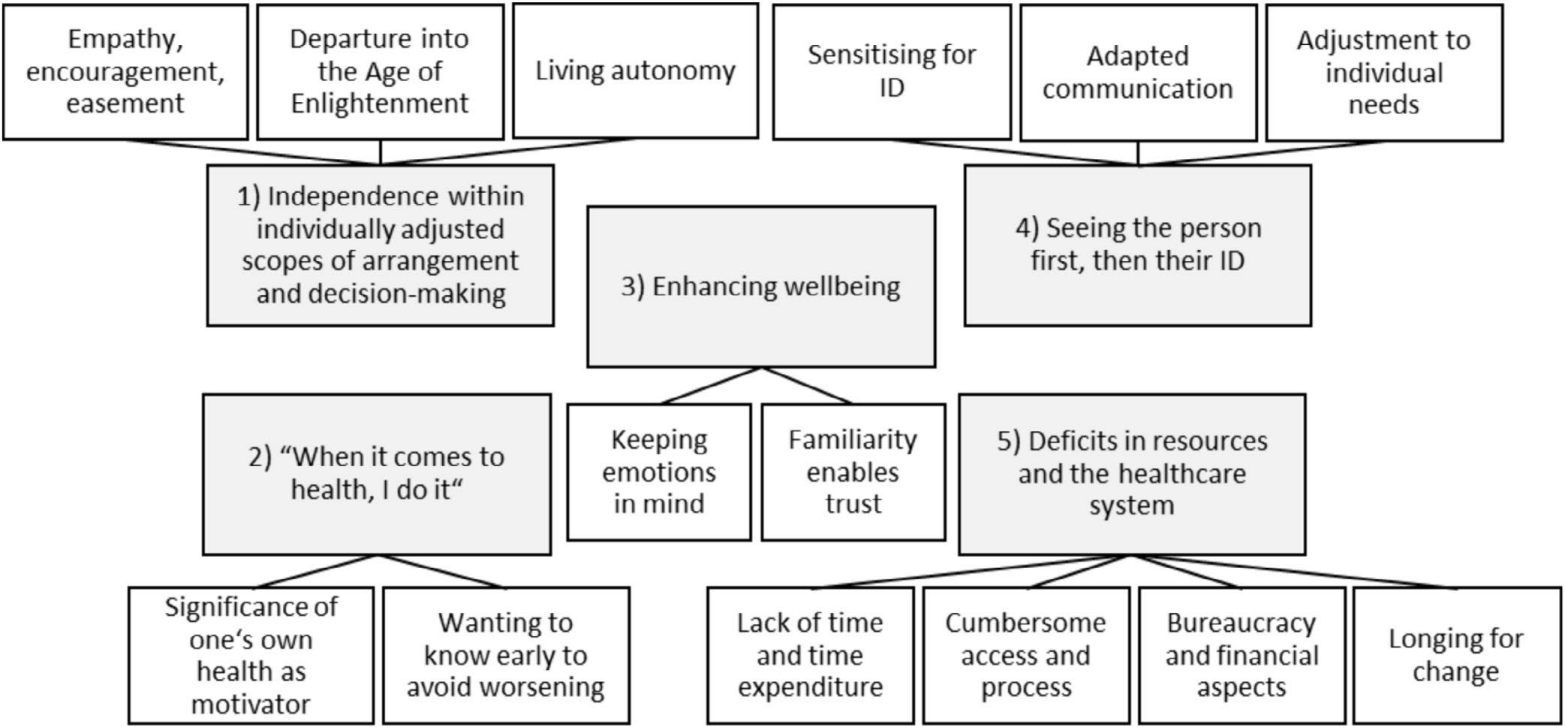
Themes and sub‐themes of the qualitative analysis.

Five themes were identified each consisting of two to four sub‐themes: (1) independence within individually adjusted scopes of arrangement and decision‐making, (2) ‘When it comes to health, I do it’, (3) enhancing wellbeing, (4) seeing the person first, then their ID, and (5) deficits in resources and the healthcare system. Figure 1 provides an overview of the themes and sub‐themes. Table 2 lists the themes and sub‐themes with codes and selected quotes. Participants' responses were translated from German into English by one team member and blindly re‐translated by another team member. The answers were then cross‐checked, adjusted and any ambiguities discussed with a third person.

**TABLE 2:** Themes and sub‐themes with codes and selected quotes.

Subtheme	Included codes	Selected quotes
Theme 1: Independence within individually adjusted scopes of arrangement and decision‐making		
Empathy, encouragement, easement	Encouragement Support to enable easier testing (Wish for) support person Support person needed Important role of caregiver/family Family over‐care Medical care is with facility Responsibility for medical care is with the facility Medical responsibility should be with GP/doctors Reminder for check‐ups/screenings Reward after doctor visit	P30: That the relatives tell her that it is good P19: For example, if you haven't done something like this before, that you say okay, I'll support you with this and I'll show you how to do it and maybe you can do it yourself next time P02: If I asked my sister and I am glad that my sister says ‘yes’ and accompanies me, then I have a person with me when I feel anxious P01: That she doesn't have to be afraid. That someone will go with her. Or that Sarah says to her parents, I don't need anyone with me P09: I and my mum went to the dentist, but I went straight into the practice on my own, because I wanted to learn how to do that. (…) P11: My parents would say ‘no, we're not doing that’. Because they always say ‘you never know what might happen’ and my parents are a bit scared P06: Mum makes the appointment, but I go alone P02: We always went to the coffee house after the doctor
Departure into the age of enlightenment	CRC Screening not a topic Lack of education Desire/need/importance for medical education Medical education should be given by doctors Need for specialised educational offers Understandability of education important More information No additional education needed (due to prior experience) Educational resources No cancer diagnosis—No cancer education by medical staff Own research necessary Lack of access to educational resources	P09: Well, that she simply explains this properly to her child (fictional person in the case study (FP)) and explains why the faecal test has to be done and what the reason is for the whole thing and yes, explains this properly. I think that's the crucial point for an examination where you say it has to fit P16: But it's just difficult because you don't understand it and they talk and talk and then you sit in front of the computer and then suddenly the caregiver finds out what it means. And that's not the job of the accommodation facility, it's the job of the doctors P27: And not everyone has a computer where you can google it, but if you're planning something like this I want to be informed. From the doctor, of course P30: That my doctor explains this P24: In any case, too little information at all for the examinations as I see it is very little widespread, even the intestinal examination if you do not have something and is pointed out. (…) So it is very difficult to get information P26: I didn't know that these tests existed. But since I had the intestinal inflammation, I knew they existed
Living autonomy	Actively looking for help Self‐determination Independence	P19: For example, she (FP) goes to the caregivers and says: ‘I don't know how this works, I don't know what to do. Can you please help me.’ (…) And whether it's the relatives or the carers or whoever is responsible, but that she can simply choose for herself. Do I now do it with a so‐called trusted person from the institution or with the parents or relatives or whoever. So that you know, she should actually decide for herself P01: Support. Explain why she should do it. For what. But Sarah (FP) is capable of deciding for herself whether she would have it done, that's her decision. But if you inform her, nothing will stand in her way and she'll say, I'll do it P29: She doesn't have to, but it's her decision afterwards. Go or don't go P21: I can't force people to do it and I shouldn't force them either. Because that doesn't help
Theme 2: ‘When it comes to health, I do it’		
Significance of one's own health as motivator	Attitude towards doctor visits Attitude towards screening Doctor visits as a must‐do Importance of own health Relation of cancer/cancer prevention and age Urgency Own experience with cancer Own experience with colon cancer CRC cases in personal environment Cancer cases in personal environment Recommendation for cancer screening by doctors Recommendation due to symptoms/suspicion	P03: I don't go to the doctor unnecessarily. Only if I have to P06: I'd almost rather go to the doctor than the dentist P18: If I have to go because of my health, I have to go, but that's not my thing. But hey P24: I'm very well. Good. With my health. I've had all the tests that are possible this year. I've also had the bowel examinations. That's also very important to me. (…) In my opinion, it should be compulsory from a certain age. It would be a great thing if everyone had to do it, because it really (…) is very important for health, of course P27: Of course I would do such a routine thing. When I get to that age and, why shouldn't I do it? P27: No, when I was young, I never used to think that there was such a thing [CRC screenings] P09: Yes, especially for your own body, when you know you won't have anything. So I don't think there's anything wrong with that [screenings] P08: If it's important, I would do it once in a while. It depends. But I'm still young. Still, you never know. It's important to have it done, to have it looked at. (O2, Pos. 450) P16: So, when it comes to my health, of course. (…)So if it's important for my health, I'm ready for any examination and any change P14: Because it's good for your health P26: If the doctor says it should be done, then it must be done P29: I had to because she [doctor] wasn't sure because I have had pain at times. She said we'd send him to do a colonoscopy
Wanting to know early to avoid worsening	Prevention to avoid pain/against worse/to help	P24: In my opinion, early detection is the best way to cure because the more advanced, the less chance you have P30: If she [FP] does this, you can see what is going on in her digestive tract and if you find anything, you can do something about it early on
	Speaking about topic perceived as interesting/important/want to be heard Low awareness of CRC screening Stool sample test preferred	P25: I would love to have a conversation like this again P01: All interesting, these topics now Interviewer: So, everything is still good? Was that disturbing or too much? P02: No, that was good. That you understand us P01: We should talk more about health. (…) The whole healthcare system isn't right. It's not being addressed P09: And I think the questions you asked us were also good, I would say. They were good P30: Nice that you were there
Theme 3: Enhancing wellbeing		
Keeping emotions in mind	Lasting (traumatic) experiences Personal environment bad experience with colonoscopy Danger Traumatic experience Personal environment participation in CRC screening Physical sensations Disgust Discomfort Tension Pain Problems with colon cleansing No pain‐experience during colonoscopy No colonoscopy due to backpain Emotional reactions Fear Fear talking about cancer Anger Relief after screening Relief watching colonoscopy live Relief/gratefulness Cognitive processes Scepticism Positive mindset Overload/overwhelming Uncertainty/sense of security Colonoscopy imagined as uncomfortable	P03: Well that's as women, of course, but (unv.) for some it's bad that they don't dare to go to the doctor because they've had bad experiences P01: First you have to cleanse your stomach, then you get a litre of what you have to drink. That's disgusting P31: Well, it [colonoscopy] will be unpleasant P06: I would find that [colonoscopy] awful P23: Because it's uncomfortable. To drink and all of it P09: So you're not happy at first when you know you have to have an examination. But when you know you haven't had anything, you might be glad that you did it P13: I would do one, but I'm perhaps far too inhibited and scared to do it P16: If it [doctor's consultation] is too much for me, that I then go out and she [caregiver] just tells me later
Familiarity enables trust	Familiarity Importance of trust Shame/Embarrassment Uncomfortable procedure (shameful/embarrassment) Feel well cared for Satisfaction with medical care Social interaction in a medical setting ○ Friendliness ○ Sense of humour/Charme	P24: I just think you have to talk to relatives, doctors, people you trust, that you try to recognise the feelings, because somehow everyone has them P16: Many doctors have known me since I was a child, which is why I have a good relationship with most of them P30: Okay, because the gynaecologist was the first gynaecologist I've ever had and she's very nice. She explains everything well. I'm in good hands there P29: All [medical staff in hospital] friendly. Everyone likes me. Everyone, including the boss. All of them P18: If you're in pain, you want it to be treated quickly and to be over quickly, and you don't want to be cold‐shouldered and nobody goes to hospital just for fun
Theme 4: Seeing the person fist, then their ID		
Sensitising for ID	Discrimination because of ID Need for inclusivity Effort ○ Need for patience during interaction (medical staff) ○ Willingness to take time ○ Listening ○ Lack of understanding/empathy of medical staff ○ Lack of action of medical staff Sending home ○ Lack of education of doctors on IB ○ Lack of knowledge of living structures of people with IB ○ Barrier – free architecture/setting/equipment ○ Specialised medical staff Lack of medical facilities	P24: The GPs are unfriendly. Most of them don't have enough staff. That's also a problem. And especially if you already have some kind of impairment, you get less information yourself. You have to take someone with you who knows more about you than you do, even though you're the one who's ill. That's a very big problem in medicine (…) Or if I go with my wife, I have an impairment and am (unclear) then he only talks to my wife, does he tolerate that, does he not tolerate that? Can he take that? And he talks to me very little. So I get almost no information myself, even though I'm the patient (…)So I think that the doctors should talk more to you as a patient P02: In my case, the doctor never told me what I had, he only ever talked to my mum P16: Thank God I can stand up, but there are people who can't stand up at all. And so you have to think carefully about how you can support people with high support needs. That's very important to me, that you always think about them. (…) that people with high support needs are also taken into consideration and that they are also treated well, just like people who have no limitations P02: That every person who wants to read it can read it. Whether they have a disability or not P22: I find the medical letters at the end very complicated, where I have no idea. It's in difficult language. The caregiver reads it for me. So for me it's also written too small. And that they just say you have to change something, but they're not in your body and don't know how difficult it is for you. And that annoys me P18: My mum takes me there. And she doesn't understand everything, but she takes me there and I explain it to her in Turkish. Then I explain what I understood. Or someone else comes with me, that's my brother. He also says what he has understood from the doctor and tells her
Adapted communication	Adapted communication in the medical context Modelling new actions/situations Tangible media Concrete open communication Directly addressing the patient	P19: Much more information would be needed in simpler language, because not everyone can understand this complicated, difficult language, the way doctors like to use it, not everyone can understand exactly what is meant. (…) It's almost like a foreign language, like specialised Chinese, you could say. So really explaining it straight away so that you understand it and not these complicated words P27: And finally, a short video where all the topics are summarised P25: If you put up lots of brochures about this disease in doctors' practices. And there are so many posters for advertising. We don't have that at all. (…) As I have seen for bowel cancer screening, information is provided with adverts on television P23: There should be pictures with smileys. There should be pictures with larger fonts and so on P22: It is in difficult language so for me the caregiver reads it P24: You mustn't forget the weak ones, because anyone who is overlooked is one too many in my opinion
Adjustment to individual needs	Comorbidities/physical issues play a role Issues/difficulties due to physical restrictions Narcosis controversial Individual adjustment to support needs	P19: I am also epileptic, so I also take medication for epilepsy and when. I'm well adjusted to my medication. And if I had an operation now, it would probably have to be readjusted. I don't know what it will be like afterwards and things like that. So I have to take a lot of things into account P06: I don't like that [narcosis] at all P24: It would have to be in the direction that it can be done by everyone, that it is understandable for everyone P16: I can stand up, thank God, but there are people who can't stand up at all. And so you have to think carefully about how to support people with high support needs
Theme 5: Deficits in resources and the system		
Lack of time and time expenditure	Late appointments/problems with appointments Own time resources limited Frequency/time consumption of visits Waiting time at the facility Limited time resources in a medical setting	P01: It doesn't just take time for people with learning difficulties. It takes time in general, yes, it takes time in all kinds of situations, you have to wait months. It's not good for your health. You can't even call it health. Health is when I get to the doctor straight away or get an appointment straight away and don't have to wait a year. (…)But if you have to go to the doctor and then you have to wait months. This increases the nervousness of people with learning difficulties. The long waiting times are very distressing and there are more feelings of anxiety P18: But the waiting is insane P23: Why I don't like going, the waiting. (unv.) The whole day is always lost P27: But that can't be the case, that's why I make an appointment. And that I'm then brushed off and blablabla
Cumbersome access and process	Distance to the doctor's Patient transport sth. to think about Be sent around in the hospital/from doctor to doctor Communication issues between different medical entities/staff Referral to specialist necessary Dissatisfaction with medical care	P26: The doctors have to talk to each other P23: You're always sent around from one thing to another It would be good if, for example, the doctor performing the operation could consult with the epilepsy outpatient clinic P24: When I go to the GP, I always have to get a referral from the specialist first because it's a specialist who does the colonoscopy. I have to go to the GP to get a referral so that he can do it
Bureaucracy and financial aspects	Bureaucratic hurdles (paperwork, external circumstances) Financial aspects in the medical context	P27: But that's madness, and my suggestion would be to save the data on the e‐card, for example. The person who inserts it practically has all the data, sees that I'm allergic, that I've already had all this blablabla. That would be a facilitation, like that P19: Yes, it's stressful for me and it's stressful for me. When I know I have an examination now, I never know what to take with me. For example, what documents P30: Which is good anyway, because it's a precaution. If I do that, my insurance company will also pay me 100 euros more P24: With me he always asks if you want one, but if you want an anaesthetic then you have to pay for it yourself. The examination is done by the health insurance company. But if you want an injection, you have to pay for it. He says only the examination is free
Longing for change	Demand for a legal obligation for screening tests Health pass All at the same place Political inactivity (lack of will to understand) Proactive call for change	P26: I just had an idea. There is the health pass. (.) And if it says that another examination is necessary for a person with high support needs and this is ticked. Then the employee knows or should know that the person needs to be examined P01: But if I'm already in hospital, for example, they could do all the examinations at the hospital, that would be even easier P21: Yes, everything there in the same house P01: Something really needs to change in the healthcare system(…)So the whole healthcare system isn't right. People just talk about it, but nothing happens P16: Maybe it's important to consider that you hire interpreters, because I had the same problem with my mum, she couldn't speak German very well and I always had to do it. And it's also difficult as a child or young person or as an adult or a person with learning difficulties to translate that you always have an interpreter on site. It would also make things easier for people

*Note:* The interviews and focus groups were conducted in German. Quotes were translated to English by the research team.

#### 3.2.1 Independence Within Individually Adjusted Scopes of Arrangement and Decision‐Making

The first theme describes that people with ID want to live as independently and self‐determined as possible. Environments and living spaces should be designed to enable and promote independence and freedom of choice. The sub‐themes describe approaches to achieving independence. First, support should be provided in form of *empathy, encouragement and easement*. One respondent described that she ‘might need someone to encourage her’ (P18). As a facilitation, participant 1 suggested a reminder: ‘No, you can write to the patient. […] You haven't been there in the last year or two. Please. It would be desirable to have an examination again’.

The second sub‐theme, *departure into the Age of Enlightenment*, summarised the expressed desire for education and information to be able to make informed decisions. Participant 9 described: ‘And above all, (…) to be informed about what exactly is going on, if you get a precise description from the doctors’. The responsibility for providing information is clearly seen as being with the physicians, ‘Well, of course, the specialists with the help of the responsible doctors who have a clue’ (P27). All information should be understandable, as participant 2 emphasised: ‘Information, information. That also means in easy language’. It was also reported that it is very difficult to get appropriate information.

The third sub‐theme, *living autonomy*, emphasises that awareness of independence, self‐determination, and autonomy should be created, implemented and respected, as expressed by participant 4: ‘I like to do it alone. Because I want to live independently’.

#### 3.2.2 ‘When It Comes to Health, I Do It’

The second theme, quoted directly from the data, and the two sub‐themes describe influences on health behaviour. In the first sub‐theme, respondents emphasised that their own health was important to them, that they were interested in their own bodies and wanted to know what was going on inside them. Mixed attitudes were expressed towards visiting the doctor, but a generally positive attitude towards CRC screening. Preventive care was seen as good and important. Maintaining one's own health was ranked as the most important motivator for their own health behaviour. Accordingly, a willingness to make unpopular visits to the doctor was described: ‘It's nice to do something else, but if it's good for something, it's certainly not wrong to do it’ (P9). The second sub‐theme summarises that people are aware of the importance of early detection of cancer, ‘In my opinion, early detection is the best way to cure, because the more advanced, the less chance you have’ (P24).

On a meta level, talking about cancer and cancer prevention was perceived as positive and important, highlighting health as an important topic for people with ID. In general, the awareness of CRC screening among all participants was low. Colonoscopies were mainly recommended by doctors based on symptoms, rather than as part of a screening.

#### 3.2.3 Enhancing Wellbeing

The third theme *enhancing wellbeing* stresses the importance of the psychological and emotional component. Doctor visits were often associated with many negative emotions and experiences, as participant 16 clarified: ‘And I always take a stress ball or something with me because I'm always nervous and I always have extreme stomach pains a few days before’. Therefore, it is particularly important to address and promote well‐being, as this has an impact on the willingness and frequency to visit a doctor and attend screenings.

The sub‐theme *keeping emotions in mind* refers to the importance of emotions particularly in relation to colonoscopy. The data revealed that stool samples (FOBT, FIT) were preferred to colonoscopies, primarily due to the daunting and often unpleasant idea of a colonoscopy. Colonoscopy was discussed as a highly emotional topic and was associated with physical sensations and emotional reactions such as disgust, pain, discomfort, fear, but also relief and gratitude afterwards. The interviews clearly showed that respondents who had already had a colonoscopy described it as not bad, whereas the idea of the examination was associated with more negative emotions in people who had not yet had a colonoscopy. On a cognitive level, scepticism, overload, excessive demands and uncertainty were mentioned.

At an interpersonal relationship level, trust is important and is reflected in the sub‐theme *familiarity enables trust*, as stated: ‘She (fictional person in the case study) would need a person, a good confidant, to explain everything to her in more detail. And then this trusted person should explain that it's for her health and that it's for her safety’ (P21). Trust can be fostered through familiarity with the environment, people and procedures, and can often be built simply through small things such as friendliness or charm, as one participant described: ‘It's clear that an examination won't be easy, but I think that if you're treated nicely there, I think the feeling you might have in front of the doctor will certainly be gone’ (P9). To feel well cared for was also often mentioned.

#### 3.2.4 Seeing the Person First, Then Their ID

People are often reduced to their ID, which is reflected in described experiences of stigmatisation and discrimination, such as ‘(…) but they [doctors] probably think to themselves, he's 100% infantile, he doesn't understand anything anyway. That's why they talk to my wife’ (P24). The sub‐themes *sensitising for ID, adapted communication*, and *adjustment to individual needs* summarise that each person can and should be addressed according to their needs.

A lack of willingness to take the time and insufficient knowledge about ID were particularly noted among medical staff. Participants emphasised that medical staff often do not know how to interact with people with ID and turn them away. Participant 26 suggested that, ‘Doctors should be educated about disability’. The qualities of effort, patience and listening were mentioned as being beneficial. The need to increase awareness and sensitivity to ID is reflected not only in poorer healthcare, but also in inadequate accessibility. Demands were expressed: ‘That every person who wants to read it, can read it’ (P2), and ‘If the lift is broken, it won't go up. (..) all doctors should be barrier‐free. Easy access like in a shop. Easy access to the doctor’ (P1). Therefore, barrier‐free accessibility tailored to the needs of people with ID and specialised medical staff was often requested.

The use of adapted communication in interaction was emphasised. Easy language, understandable expressions, and direct, open communication were desired, as stated: ‘And especially if you already have some kind of impairment, you get less information yourself. You must take someone with you who gets more information about you, even though you are the person who is ill’ (P24). Patience, slow explanations, video clips, illustrations and the use of larger print, numbers and Braille were mentioned as helpful. Individual support needs, comorbidities, intersectionality, physical limitations and anaesthesia were widely discussed in the interviews and focus groups, emphasising that adjustment to individual needs is important.

#### 3.2.5 Deficits in Resources and the Healthcare System

The last theme describes the deficits in the healthcare systems. Deficits were categorised into the sub‐themes of *lack of time and time expenditure*, *cumbersome access and process*, *bureaucracy and financial aspects*. This led to a strong desire for change captured in the sub‐theme *longing for change*, with politicians being called upon, as: ‘So the whole healthcare system isn't right. People talk about it, but nothing happens’ (P2). Finally, participant 24 emphasised that change is only possible through joint commitment and awareness raising: ‘That is important because you [focus group leader] are people where change can perhaps be forced. Where we are less able to. I also think it makes sense to do something like this and I think it's great, there are certainly many points where you can achieve change. Thank you’.

## 4 Discussion

Our study provides first insights into the perspectives of people with ID on their experiences of CRC screening as a health prevention measure. The results show that health is important to people with ID. Screening and preventive measures are rated as useful. With regard to low CRC participation rates, the data revealed that people with ID face significant challenges and barriers in accessing healthcare and screening services.

The discrimination and stigmatisation described by the participants is consistent with studies showing that people with ID are among the most stigmatised and marginalised groups in society [26, 27, 28]. It is essential that healthcare systems and public health campaigns work to eliminate discrimination and increase accessibility, with the aim of providing equitable healthcare for all. Campaigns raising awareness for screening programmes should use accessible formats, such as easy language and visual aids to ensure everyone is aware of the programme. Accessible, barrier‐free invitation letters (e.g., easy language, visual aids, clear explanations of steps to be taken) as reminders and educational booklets are important facilitators, as the data indicate. A system to identify non‐responders were further suggested to improve screening participation [12]. Organised screening programmes should also take into account recommendations for at‐risk groups and consider lowering the recommended age of participation, especially for people with ID who may have accelerated ageing [5, 6].

As limited access and availability of appropriate information and educational materials was identified as a major barrier, accessible information, using easy language and visual aids should be widely available, with information seminars for individuals with ID and their caregivers. Supportive frameworks, environments and situations to talk about these topics, as well as clear, accessible information can empower individuals with ID in their healthcare decisions to promote a self‐determined and independent life [25, 29, 30].

Although this was not the primary research question, our results point to an alarming lack of trained health professionals to provide adequate care for people with ID. Many participants reported disturbing experiences. Shortcomings are reflected in negative attitudes and behaviours, insecurities, lack of knowledge about ID, lack of experience, lack of appropriate support, reluctance to adapt and inadequate communication skills. Similar challenges have been identified in broader assessments of the healthcare system [27]. This is particularly concerning as medical staff are perceived by respondents to have a significant influence on access to and outcomes of healthcare. Mandatory ID‐specific training for health professionals would be essential to improve care quality for people with ID and to promote cancer symptom recognition. This is particularly important given that diagnostic overshadowing and ableism are common issues [8, 31].

Regarding possible cognitive deficits as a barrier to screening mentioned by other studies [12, 25], the study underlines the importance of the emotional and interpersonal level. Feeling well cared for has a significant impact on screening participation and may be more influential than the cognitive level. The data also underlines the need to address individuality through a person‐centred approach and to enhance the emotional and interpersonal aspects, e.g., well‐being, trust, security and stability.

As a general lack of ID‐appropriate healthcare services was identified. The described deficits in the healthcare system affect people with ID more frequently and more severely than those without ID, causing multiple burdens and discrimination. To address systemic and organisational deficits, inclusive health infrastructures that meet different support needs are required. Accessibility is crucial, including barrier‐free access to health services, standardisation of screening, cost‐free screening/narcosis, availability of interpreters, accessibility of transport, accessibility of texts and forms. Organisationally, attention should be paid to inclusive and equitable frameworks, such as allocating more time for appointments, enabling short waiting times, reading out names in addition to the digital display, and less bureaucracy by providing forms in advance, storing standard information on the e‐card or using health passports that document a person's abilities, needs, general state of health and treatment preferences [32]. In this regard additional specialised healthcare services tailored to the needs of people with ID are suggested. Finally, societal awareness and inclusive community interactions are vital to reducing stigma and fostering acceptance for individuals with ID.

### 4.1 Limitations

This study has several limitations. First, while the sample was diverse, it excluded individuals with severe ID, focusing primarily on those with mild ID, who comprise approximately 80% of the ID population. Second, the study was constrained by limitations in existing research methodologies. Whereas focus groups are an effective method for gathering data on the experiences and actively involving people with ID in research [17, 19], there is limited guidance on conducting interviews and designing accessible survey tools specifically for individuals with ID. Furthermore, the interview guide was developed, and the data analysis conducted without the direct input from individuals with ID or the public, which may have limited the study's ability to fully capture their perspectives. Lastly, although the study aimed to employ participatory research methods and involved people with ID in reviewing the results, full participatory engagement was not achieved.

## 5 Conclusion

**FIGURE 2:**
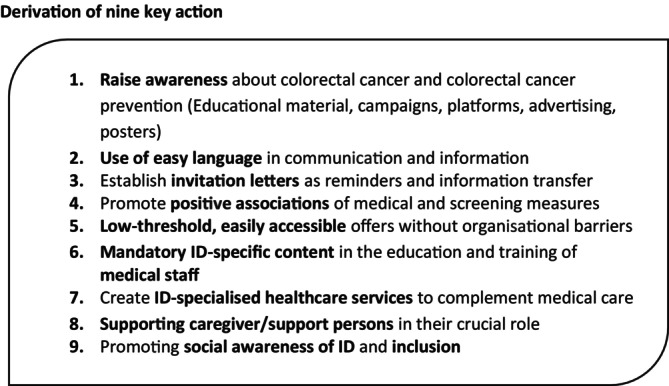
Derivation of nine key action.

The findings of this study highlight the lack of inclusivity in healthcare and underline the necessity to implement inclusive, accessible CRC screening programmes that address the needs of individuals with ID. Several actionable steps can be derived from the results and are presented in Figure 2. As required by the UN‐CRPD, good health should be an achievable right for all, yet current health systems often fail to provide equitable opportunities for people with disabilities, including ID. Moving towards systemic equity will require structural changes to ensure that everyone has a real chance to maintain good health and achieve personal fulfilment. A key takeaway from this research is the importance of fostering a sense of safety for people with ID, particularly during procedures such as colonoscopies, which are essential for cancer prevention but can be daunting. By approaching healthcare interactions with openness and respect, and by considering the individual's ID only as a secondary consideration, healthcare providers can better meet the needs of these patients and support their engagement in preventive health practices. The key considerations identified in this study can inform the development of inclusive screening programmes, thereby facilitating equitable healthcare for all.

## Author Contributions

**Theresa Wagner:** data curation, formal analysis, investigation, project administration, visualisation, writing – original draft, writing – review and editing. **Alma R. Herscovici:** data curation, formal analysis, writing – review and editing. **Amelie Fuchs:** data curation, formal analysis, writing – review and editing. **Sebastian Kabas:** data curation, formal analysis, writing – review and editing. **Mara Hilbert:** data curation, formal analysis, writing – review and editing. **Laura M. König:** formal analysis, investigation, supervision, writing – review and editing. **Matthias Unseld:** conceptualization, investigation, methodology supervision, writing – review and editing. **Elisabeth L. Zeilinger:** conceptualization, formal analysis, funding acquisition, investigation, methodology, project administration, supervision, writing – original draft, writing – review and editing.

## Ethics Statement

The study was approved by the Ethics Committee of the Medical University of Vienna (No. 2125/2022).

## Conflicts of Interest

The authors declare no conflicts of interest.

## Supporting information


**File S1:** cam471212‐sup‐0001‐FileS1.docx.

## Data Availability

The authors have nothing to report.
