# Supplementary material for: Towards Inclusive Colorectal Cancer Screening: Experiences and Needs of Adults With Intellectual Disabilities
Source: Cancer Med. 2025 Sep 4;14(17):e71212. doi: 10.1002/cam4.71212 (PMC12409469; doi:10.1002/cam4.71212)
Supplement: Supplementary file 1 — File S1: cam471212‐sup‐0001‐FileS1.docx. [file CAM4-14-e71212-s001.docx]

## Supplementary File

**Topics of final interview guideline and materials used**

| **Topics** | **Material** |
| --- | --- |
| **1. Welcome, introduction and organisational process** (informed consent, getting to know each other, collecting demographic data, current wellbeing) | Flipchart with timeline, presentation, sheets, pictures, pictograms, smilies, voting |
| **2. Thematic introduction** |  |
| Questions about health, doctor visits and feelings; Questions about cancer, colorectal cancer, knowledge and information about it wanted  E.g. Do you know what colorectal cancer is?  What do you associate with colorectal cancer?  Would you like (more) information? | Presentation, pictures, pictograms |
| Explanation of cancer, colorectal cancer and the importance of detecting cancer early | Presentation, pictograms, pictures |
| **3. Early detection** |  |
| Questions about colorectal cancer screening, knowledge and information about it wanted  E.g. Do you know what you can do to detect colorectal cancer early?  Do you feel you know enough about screening (why yes/no)? | Presentation, pictures, pictograms |
| Explanation of examinations, incl. faecal test and colonoscopy | Pictograms, pictures, materials from ‘Simply talking about health and illness’ and METACOM |
| **4. Colorectal cancer screening** |  |
| Questions about attitudes towards screening tests and experience with faecal test and/or colonoscopy  E.g. What do you think of early detection tests?  Have you ever had a faecal test?  Can you tell us what it was like? | Presentation, pictograms, pictures |
| **5. Emotions and feelings during these examinations** |  |
| Questions about emotions, problems, feelings during the examination and willingness to repeat the examination  E.g. How did you feel during the examination?  Were there any problems?  What was helpful to you? | Smilies, voting |
| **6. Support requirements** |  |
| Questions for accompaniment, support, explanations and wishes  E.g. Who was with you during the test?  How were the medical staff?  What do you wish you could have had? | Presentation, pictograms, pictures |
| **7. If no screening has been done / recall problems** |  |
| Questions about willingness to have a faecal test, colonoscopy and barriers, facilitators, emotions, needs  E.g. Would you have a colonoscopy? (Why yes/no)  What would you need? | Voting, smilies, pictograms |
| **8. Case study** Description of Sarah, a 48-year-old woman with intellectual disabilities, who is unsure about having a faecal test | Pictures, pictograms |
| Questions for advice for Sarah, relatives, doctors, and needs  E.g. What advice would you give to Sarah?  What do you think Sarah needs?  What would you like to tell Sarah's doctors? | Presentation, pictures, pictograms |
| **9. Summary and conclusion** |  |
| Brief summary of the topics, positive conclusion, current wellbeing | Pictures, pictograms, smilies |

*Note.* All topics, questions and materials were prepared in easy language (language level A1-A2). Current state of wellbeing was regularly asked during the interviews/focus groups. Material used was from the Monique brochure of the Association ONCODEFI which is available in German (https://www.lebenshilfe.de/informieren/familie/krebsinformationen).
